# CO-PRODUCED IDEAS FOR SMART HOME TECHNOLOGIES TO SUPPORT ENGAGEMENT IN MEANINGFUL ACTIVITIES

**DOI:** 10.1093/geroni/igae098.2557

**Published:** 2024-12-31

**Authors:** William Son Galanza, Steven Schmidt, Oskar Jonsson, Jens Offerman, Nebojsa Malesevic, Susanne Iwarsson, Sofi Fristedt

**Affiliations:** Lund University, Lund, Skane Lan, Sweden; Lund University, Lund, Skane Lan, Sweden; Lund University, Lund, Skane Lan, Sweden; Lund University, Lund, Skane Lan, Sweden; Lund University, Lund, Skane Lan, Sweden; Lund University, Lund, Skane Lan, Sweden; Lund University, Lund, Skane Lan, Sweden

## Abstract

While research in the field of technology is surging, research on older adults playing an active role in designing and developing smart home technology (SHT) to enhance usability and adoption is lacking. It is important to co-develop products with potential users to better match their needs and desires. This study aims to generate ideas about SHT that can enhance the use and adoption of SHT that can support engagement in meaningful activities while ageing. The research circle methodology was utilized to elicit co-produced ideas for SHT and their functions with participants from different generations (30-39, 50-59 and 70-79 years old; n=9), together with two professionals with expertise in SHT and four researchers. The strength of research circle methodology is knowledge translation through the collaborative effort of participants. The findings revealed that the participants preferred SHT which can support independence, engagement in social experiences, and participation in meaningful activities while ageing, rather than smart home appliances or full home automation. Moreover, SHT which can create a stimulating environment to positively impact physical, cognitive, and psychosocial factors were highlighted. Some ideas generated were multi-purpose functions and a system that can analyze patterns for monitoring activities and prevention of disability or its progression. Non-technology support such as informal carers and neighborhood and community support were also prioritized. Our study highlights the benefits of collaboration among diverse stakeholders (older adults, informal carers, technology professionals and researchers) to generate ideas about preferences for SHT that can better support the needs and desires of older adults.

